# An Absorption-Based PDMS/f-CNT Mass-Capacitor for Continuous Monitoring of Fire-Derived Organic Contamination

**DOI:** 10.3390/s26165220

**Published:** 2026-08-18

**Authors:** Negar Heidari, Morteza Ghafar-Zadeh, Azadeh Amrollahi, Parviz Norouzi, Sebastian Magierowski, Ebrahim Ghafar-Zadeh

**Affiliations:** 1 Biologically Inspired Sensors and Actuators (BioSA) Laboratory, Department of Electrical Engineering and Computer Science (EECS), Lassonde School of Engineering, York University, North York, ON M3J 1P3, Canada; heidar3@yorku.ca (N.H.);; 2 EMIL Lab, Department of Electrical Engineering and Computer Science (EECS), Lassonde School of Engineering, York University, North York, ON M3J 1P3, Canada; karam23@my.yorku.ca (M.G.-Z.);

**Keywords:** mass-capacitor, PDMS/f-CNT composite, fire-derived contamination, sorptive sensor, capacitive sensing, cumulative exposure monitoring, firefighter exposure

## Abstract

Firefighters are exposed to complex smoke containing volatile, semivolatile, aromatic, and particulate-associated organic contaminants that accumulate on skin, clothing, and protective equipment. Conventional gas sensors monitor only selected airborne species, while passive samplers require laboratory analysis and cannot provide continuous exposure assessment. To address this limitation, we developed a proof-of-concept mass-capacitor that integrates contaminant absorption and electrical sensing within a single polydimethylsiloxane/functionalized carbon nanotube (PDMS/f-CNT) composite coated on interdigitated electrodes (IDEs). Unlike conventional sensors that report the instantaneous concentration of selected compounds, the proposed platform functions as both a sorptive collector and an electrical transducer. It continuously converts contaminant uptake, retention, and release into a time-resolved electrical response. For firefighter applications, this mass-capacitor bridges the gap between passive sorptive samplers and conventional real-time gas sensors. It enables continuous tracking of the cumulative sorbed contamination burden without requiring offline laboratory analysis or restricting the measurement to selected airborne species. A custom potentiostat applied staircase cyclic excitation, while Fast Fourier Transform (FFT)-assisted processing enabled extraction of the differential charge, ΔQ as an indicator of contaminant loading. The sensor was evaluated using smoke generated from cotton, paper, wood, and synthetic fibers. Compared with PDMS alone, the PDMS/f-CNT composite produced an approximately 24-fold higher response with minimal humidity interference (≈80% RH). Limits of detection ranged from 0.08 to 0.26 ppm, while repeated smoke exposures produced stepwise increases in ΔQ, demonstrating continuous tracking of cumulative contaminant loading. These results establish the feasibility of the mass-capacitor concept and introduce a new approach for real-time monitoring of the accumulated organic contamination burden rather than the instantaneous concentration of individual airborne compounds.

## 1. Introduction

Firefighters are repeatedly exposed to complex mixtures of combustion-derived contaminants, including volatile organic compounds, polycyclic aromatic hydrocarbons, aldehydes, and particulate matter, many of which are carcinogenic or mutagenic [1,2,3]. Reflecting this evidence, the International Agency for Research on Cancer placed occupational exposure as a firefighter in Group 1, indicating that it is carcinogenic to humans [2,4]. Canadian occupational-health reports indicate that cancer accounts for nearly 85% of accepted work-related fatality claims among firefighters. Furthermore, an Ontario cohort reported a 23% higher overall cancer incidence among firefighters compared to other workers [5,6,7]. These findings demonstrate that, despite the use of respiratory protection, turnout gear, and decontamination procedures, carcinogenic exposure remains a major occupational-health concern [8,9,10].

Fireground contaminants can enter through both inhalation and dermal routes, but exposure is not limited to compounds that remain airborne [8,11,12]. More volatile species tend to remain in the gas phase, whereas semivolatile and particle-associated organics can partition among vapor, smoke aerosol or soot, and exposed surfaces [12]. Once deposited, these contaminants may accumulate on skin and turnout gear, penetrate through or around protective layers, and contribute to continued exposure through direct contact or later off-gassing [11]. Studies of Ottawa firefighters detected dermal polycyclic aromatic hydrocarbon (PAH) contamination together with increased urinary PAH metabolites and urinary mutagenicity [3]. Controlled-fire studies similarly reported increased PAH and Volatile Organic Compound (VOC) biomarkers despite the use of self-contained breathing apparatus and full protective ensembles, supporting a contribution from dermal and surface-associated uptake [8,12,13]. Thus, contamination that partitions from smoke and is absorbed and retained by exposed surfaces represents an important component of firefighter exposure alongside airborne gases.

Despite its importance, relatively little attention has been devoted to continuously assessing the fraction of fire-derived contamination that is absorbed and retained on exposed surfaces. Most fire-exposure studies instead collect airborne contaminants using pumps, sorbent tubes, filters, or whole-air sampling and analyze them offline using gold-standard techniques, such as gas chromatography–mass spectrometry (GC–MS) [14,15,16,17]. Although chemically accurate, these methods are time-integrated and cannot track exposure continuously during a fire incident; they may also underrepresent contaminants that rapidly partition onto particles, soot, skin, gear, and other surfaces [15,16]. To provide real-time measurements, several studies have developed low-cost electrochemical gas sensors; however, these systems are generally designed for individual gases or limited analyte classes, such as CO, NO_2_, formaldehyde, selected VOCs, or individual PAHs [18,19,20,21]. They therefore monitor only selected airborne species rather than the cumulative contamination sorbed by exposed surfaces. This gap highlights the need for a real-time sorptive sensor capable of retaining and continuously measuring the surface-associated fraction of fire-derived exposure.

Among the relatively few studies addressing contamination absorbed and retained on exposed surfaces in firefighting settings, PDMS has been used as a passive sorbent for sample collection followed by GC–MS analysis. Silicone-based samplers, particularly PDMS wristbands worn inside and outside protective clothing, have captured PAHs and other organic contaminants from complex fire environments [22,23,24,25]. However, this approach requires multistep, complex, and time-consuming extraction and sample preparation before GC–MS analysis [22,26,27]. The additional need for expensive instrumentation means that the technique is available mainly in specialized laboratories. This limits real-time assessment of accumulated contamination and delays immediate actions to reduce exposure during high-contamination events. These limitations highlight the need for an electronically readable sorptive platform that combines passive accumulation with continuous measurement. Such a system can function as a sorptive mass-capacitor, temporarily retaining surface-partitioning contaminants while translating uptake-induced changes in dielectric properties into a real-time signal, thereby enabling continuous monitoring of contaminant accumulation and desorption [28].

**Figure 1:**
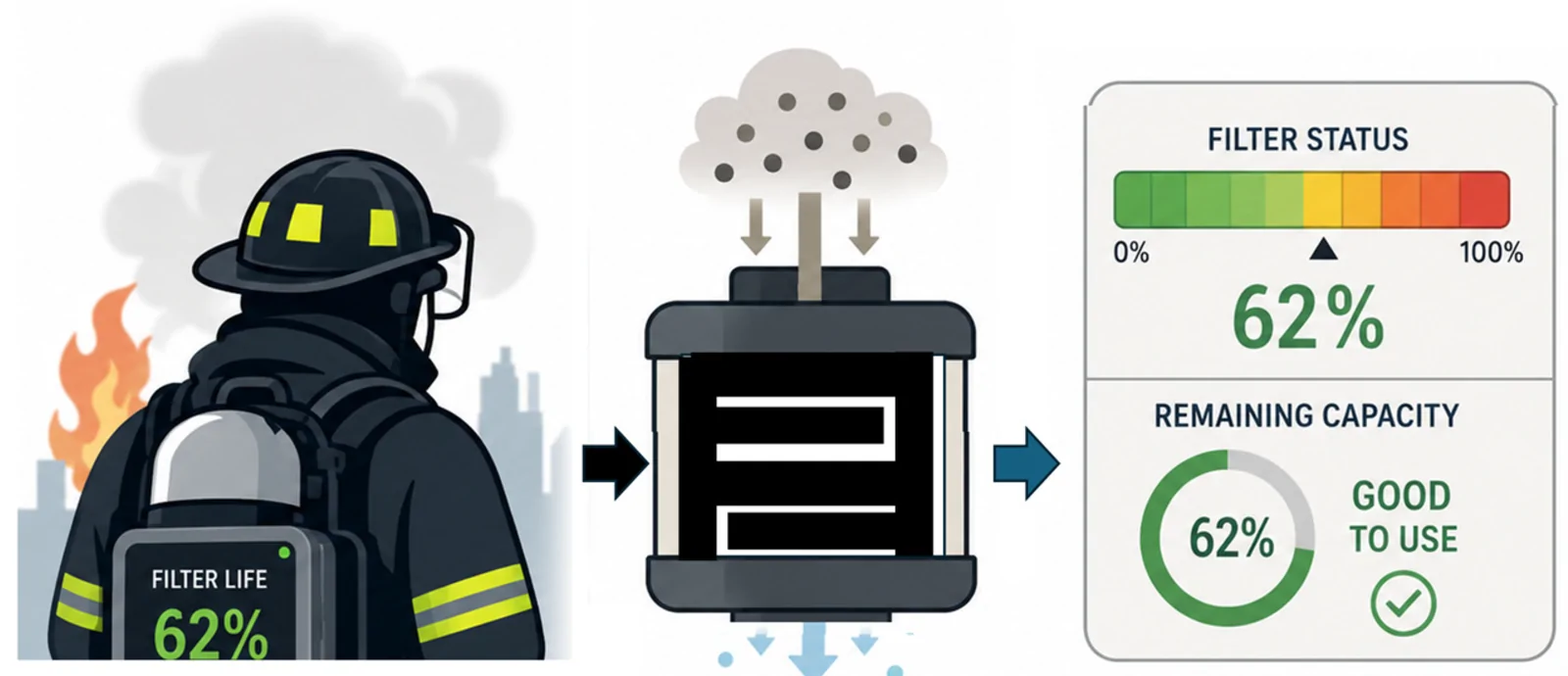
Conceptual illustration of the envisioned mass-capacitor application for firefighter protection. Fire-derived organic contaminants are collected by a sorptive sensing element, and the cumulative electrical response is translated into indicators for estimated filter status and remaining capacity.

In this work, we take a step toward a sorptive sensing approach based on a mass-capacitor, which enables the collection and temporary retention of fire-derived contaminants while translating their accumulation and release into a real-time capacitive response. This concept provides a foundation for monitoring the accumulated contamination burden rather than only the instantaneous gas-phase concentration. As conceptually illustrated in Figure 1, the mass-capacitor could ultimately be integrated with firefighter protective equipment to continuously track cumulative contaminant loading and translate the measured response into an estimated filter status or remaining capacity indicator. In the future, the mass-capacitor approach may be extended toward selective detection of specific toxic compounds using multiple tailored sorptive phases and controlled desorption. Inspired by gas chromatography, compounds could be differentiated according to their relative affinity for each sorptive material and their characteristic retention or release kinetics [28,29]. Section 2 presents recent developments in portable fire-derived gas sensing and the challenges associated with monitoring contaminants absorbed on exposed surfaces. Section 3, Section 4 and Section 5 describe the materials and methods, results and discussion, and conclusions, respectively.

## 2. Related Works

Table 1 compares representative portable or miniaturizable sensing platforms developed for monitoring fire-relevant gases and vapors. Most reported devices are designed to detect contaminants that remain in the airborne phase and employ sensing layers tailored to a specific compound or a narrow chemical class. Examples include single-walled carbon nanotube (SWCNT) networks for NO_2_ and nitrotoluene [18], amino-functionalized multi-walled carbon nanotubes (MWCNTs) for formaldehyde [19], poly(ethylene glycol) (PEG) MWCNT composites for selected VOCs [20], metal–organic framework (MOF)-coated capacitive IDEs for humidity and VOC monitoring [30], and functionalized optical sensors for naphthalene vapor [21]. Although these systems provide sensitive detection of their intended analytes, their selectivity toward individual compounds or limited vapor groups may restrict their ability to represent the overall contaminant burden generated during a fire.

In firefighting environments, a substantial portion of semivolatile and higher-molecular-weight organic contaminants may associate with soot, condense as the smoke cools, or accumulate on comparatively cooler surfaces, including firefighters’ skin and protective equipment [8,17,31]. Consequently, gas sensors that respond primarily to freely airborne compounds may underrepresent the cumulative contamination absorbed and retained on exposed surfaces. This limitation motivates the development of a sorptive sensing platform capable of collecting the hydrophobic and condensable fraction of fire-derived contamination and monitoring its accumulation over time.

As discussed in Section 1, PDMS is well suited for this purpose because of its high gas permeability and strong partition affinity toward hydrophobic organic compounds, including aromatic VOCs and PAHs [32,33,34]. Compared with permanent gases such as N_2_, condensable hydrocarbons and aromatic compounds sorb more strongly into PDMS, so the response is expected to be weighted toward the hydrophobic organic fraction of smoke. Its hydrophobic nature also helps limit interference from water vapor. PDMS has also been used as a sorptive coating in capacitive IDE-based VOC sensors, supporting its compatibility with miniaturized electrical transducers [28,35]. However, the low electrical conductivity of PDMS limits direct signal transduction, particularly when the coating is relatively thick or when absorbed molecules are located beyond the strongest electric-field region of the IDE.

Previous PDMS/CNT composite sensors demonstrated the benefits of combining the sorptive properties of PDMS with the electrically responsive properties of carbon nanotubes. PDMS/nanocarbon chemiresistors have produced real-time and reversible responses to hazardous aromatic vapors, including benzene, toluene, ethylbenzene, and xylene [36]. In addition, PDMS-coated CNT networks have demonstrated stable long-duration gas monitoring under highly humid and acidic conditions [37]. Nevertheless, these platforms were primarily evaluated using selected gas-phase compounds or gastrointestinal gas mixtures and employed resistance-based transduction rather than monitoring the time-dependent charge-storage response associated with cumulative uptake of complex fire-derived contamination.

In the proposed platform, functionalized CNTs are dispersed throughout the PDMS matrix to improve nanotube dispersion, electrical connectivity, and interfacial contact within the polymer. The graphitic CNT surfaces can also promote hydrophobic and π–π interactions with aromatic contaminants [36,38,39,40]. The resulting composite combines PDMS-based contaminant accumulation with f-CNT-assisted electrical transduction, interfacial polarization, and charge redistribution near the IDE surface. The distributed CNT network also extends the electrically responsive region through a greater portion of the polymer layer, allowing absorbed molecules located farther from the IDE surface to contribute to the measured response.

The continuous cyclic voltammetry method records the integrated charge variation, ΔQ, throughout exposure and recovery. Rather than measuring only an instantaneous resistance or capacitance value, the method follows the evolution of charge storage associated with contaminant uptake, progressive accumulation, saturation, retention, and desorption. It therefore provides information on both the magnitude and kinetics of contaminant uptake and release within the PDMS/f-CNT Mass Capacitor layer.

The present study represents an initial proof of concept toward a portable mass capacitor for monitoring cumulative contamination during fire incidents. A closed sensing chamber was used to control the introduced smoke concentration and establish the functionality, sensitivity, and repeatability of the sensing concept. Further development is required to evaluate the platform under open-environment conditions involving variable temperature, humidity, airflow, soot loading, and other harsh fireground factors. The low-power continuous cyclic voltammetry readout may also be miniaturized in future work for portable or wearable implementation.

**Table 1:** Comparison of representative sensing platforms.

Application	Sensing Layer	Readout	LOD	Ref.
NO_2_ and nitrotoluene	SWCNT network on IDE	Chemiresistive	44 ppb	[18]
Formaldehyde	Amino-functionalized MWCNTs	Chemiresistive	20 ppb	[19]
Selected VOCs: isopropanol, acetone, and isoprene	Layer-by-layer PEG/MWCNT composite on IDE	Chemiresistive	7.946–8.613 ppm	[20]
Humidity and VOCs	Cu(bdc)·xH_2_O MOF thin film on IDE	Capacitive	Not reported	[30]
Naphthalene vapor	γ-Cyclodextrin-functionalized planar Bragg grating	Optical/refractive-index	0.48 ± 0.05 ppm	[21]
Hazardous aromatic hydrocarbons	PDMS/CNT and PDMS/nanocarbon-black composites	Chemiresistive	Not reported	[36]
Complex gases under highly humid and acidic conditions	PDMS-coated SWCNT network	Chemiresistive	Not reported	[37]
Total volatile organic compounds in air	Metal-oxide-semiconductor and Total VOC sensor integrated into a miniaturized wireless monitoring system	Real-time aggregate Total VOC response with Artificial Neural Network-based baseline compensation	Not reported	[41]
Smoke from four standardized test fires and cigarette smoke	Eight-element conducting-polymer sensor array	Electronic-nose response with Principal Component Analysis discrimination	Not reported	[42]
Smoke from 16 burning materials	Six-element SnO_2_-based metal-oxide semiconductor sensor array with particulate-matter sensing	Multisensor smoke fingerprint with pattern-recognition classification	Not reported	[43]
Whole organic contamination from cotton, fiber, paper, and wood smoke	PDMS/f-CNT composite on IDE	Time-resolved cyclic voltammetry (CV) response, ΔQ	0.08–0.26 ppm	Current work

## 3. Materials and Methods

### 3.1. Materials and Reagents

Wood, paper, fiber, and cotton were selected as representative fuel materials for producing chemically complex fire-derived mixtures. Polydimethylsiloxane (PDMS; Sylgard™ 184, Dow Corning, Midland, MI, USA) was used as the sorptive polymer, and multi-walled carbon nanotubes (MWNTs; >98% carbon, 6–13 nm diameter, 2.5–20 µm length; Sigma-Aldrich, St. Louis, MO, USA) were used as conductive nanofillers. Potassium persulfate (K_2_S_2_O_8_, ≥99.0%; Merck, Darmstadt, Germany), potassium hydroxide (KOH, ≥85%; Sigma-Aldrich, St. Louis, MO, USA), ethanol (≥99.9%; Supelco, Bellefonte, PA, USA)), acetone (≥99.5%; Merck, Darmstadt, Germany), and deionized water (18.2 MΩ·cm) were used for CNT functionalization, electrode cleaning, and removal of the original polymer coating. Commercial HS-20 model humidity sensors (Amphenol Advanced Sensors, St. Marys, PA, USA), which contain interdigitated electrodes beneath a hydrophilic sensing layer, served as the base transducers. An analytical balance (Sartorius Lab Instruments GmbH & Co. KG, Göttingen, Germany; ±0.0001 g), a Cimarec^®^ hot plate (Thermo Scientific, Waltham, MA, USA), a VCX500-110V probe ultrasonic processor (Sonics & Materials, Inc., Newtown, CT, USA), and a Welch WOB-L^®^ vacuum pump (Welch Vacuum, Niles, IL, USA) were used for sample preparation, combustion, coating preparation, and chamber regeneration. All reagents were used without further purification.

### 3.2. Sensor Fabrication

#### 3.2.1. CNT Functionalization

To improve nanotube dispersibility and establish a uniformly distributed, electrically responsive network within the PDMS matrix, MWNTs were oxidatively functionalized using potassium persulfate. Forty milligrams of MWNTs were dispersed in 50 mL of deionized water by ultrasonication for 60 min at 22 °C. Potassium persulfate (0.45 g) was then added, and the pH was adjusted to 13 using concentrated KOH. The suspension was refluxed at 85 °C for 3 h under vigorous stirring. Sulfate radical anions (SO_4_•^−^) generated under alkaline conditions introduced oxygen-containing functionalities, primarily hydroxyl (–OH), carbonyl (C=O), and carboxyl (–COOH) groups, onto the nanotube walls [44]. These functionalities increased the chemical heterogeneity of the CNT surface and provided complementary polar interaction sites for substituted or partially oxidized aromatic VOCs, while the remaining graphitic regions retained dispersive, hydrophobic, and π–π interactions with nonpolar aromatic VOCs and PAHs [45,46]. The introduced functionalities also reduced CNT bundling and agglomeration, thereby promoting a more uniform distribution of f-CNTs and increasing the accessible CNT–polymer interfacial area [47]. The reaction mixture was subsequently cooled, filtered, washed repeatedly with deionized water and ethanol until neutral pH, and vacuum-dried at 60 °C for 12 h. Although oxidation increased the polarity of the CNT surface, f-CNTs constituted only 0.3 wt% of the composite. Therefore, PDMS remained the continuous phase governing the macroscopic hydrophobic character and the bulk partitioning of hydrophobic and condensable fire-derived organic contaminants. Previous studies have similarly shown that f-CNT/PDMS composites can retain strongly hydrophobic surfaces despite the presence of oxygen-functionalized CNTs [48], while PDMS exhibits high partition affinity toward hydrophobic aromatic compounds, including PAHs [49]. The distributed f-CNT network primarily provided conductive and polarizable interfaces that extended the electrically responsive volume of the otherwise insulating PDMS layer and enabled sorption-induced changes in dielectric permittivity, interfacial polarization, and charge distribution to be translated into measurable charging-current and differential-charge responses [50,51].

#### 3.2.2. Electrode Preparation and Composite Coating

**Figure 2:**
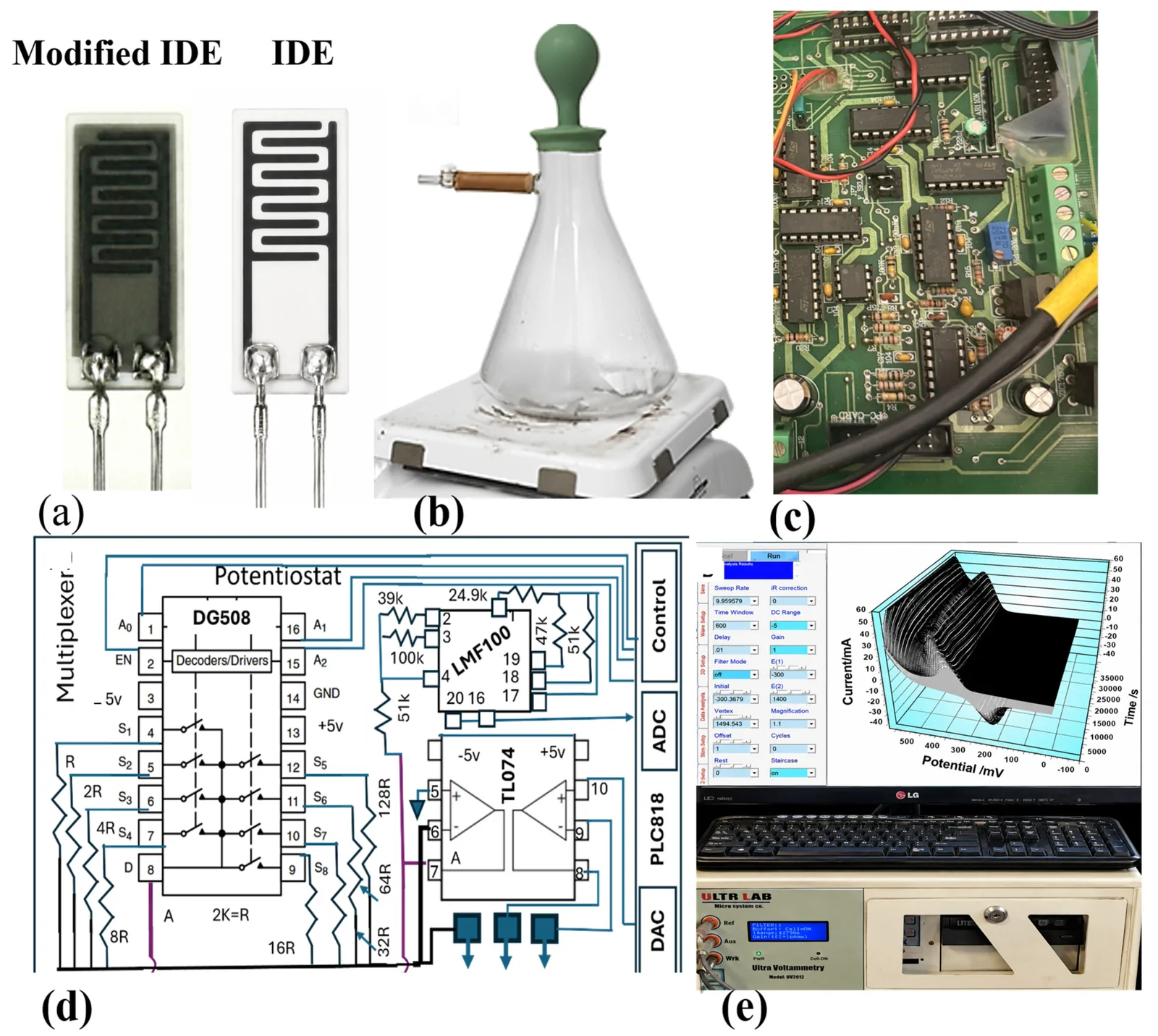
Experimental components of the smoke-sensing platform: (**a**) unmodified and PDMS/f-CNT-modified interdigitated electrodes (IDEs); (**b**) controlled smoke-generation system; (**c**) photograph of the custom electronic readout circuit; (**d**) circuit diagram of the signal-generation, amplification, filtering, and data-acquisition system; and (**e**) integrated measurement setup with the control software and real-time response visualization.

The original hydrophilic layer was removed from the 5 mm × 5 mm, 10-finger IDEs by immersion in high-purity ethanol followed by 15 min of ultrasonication. The electrodes were rinsed with deionized water for 3 min and dried with filtered air. PDMS base and curing agent were mixed at a 10:1 mass ratio, after which 0.3 wt% functionalized CNTs were incorporated by 10 min of ultrasonication. The mixture was vacuum-degassed for 10 min, deposited through a 250 µm shadow mask, and cured at 110 °C for 27 min. The mask controlled the nominal film thickness and reduced coating-to-coating variation. The selected f-CNT loading provided measurable electrical enhancement while retaining uniform dispersion; higher loadings promoted aggregation and nonuniform films, whereas lower loadings produced weaker responses. Unmodified IDEs and PDMS-only-coated IDEs were prepared as controls. Figure 2a shows the unmodified IDE and the PDMS/f-CNT-modified interdigitated electrode**.** Before repeated measurements, the sensors were rinsed with ethanol and deionized water and then air-dried. The electrodes were rinsed with ethanol and deionized water and subsequently heated at 60 °C for 15 min to ensure complete drying before deposition of the PDMS/f-CNT sensing layer. 

### 3.3. Controlled Smoke Generation System

A controlled smoke-generation system was constructed from a 1000 mL borosilicate filtering flask with a 10 mm side outlet (Figure 2b). The neck was sealed with a latex membrane, and the flask was positioned horizontally on a Cimarec^®^ hot plate maintained at 600 °C. Wood, cotton, fiber, and paper were combusted separately for 5 min under the same thermal conditions. The initial fuel mass, m_f_, and residual mass after combustion, m_r_, were recorded with four-decimal-place precision. The consumed mass was calculated as Δm = m_f_ − m_r_. Replicate burns were accepted when mass loss remained within ±5%, providing a practical check on smoke-generation consistency.

(1) $$\Delta m=m_{f}-m_{r}$$

Immediately after combustion, a defined volume of the generated atmosphere, ΔV, was withdrawn through the side outlet with a 50 mL gas-tight syringe. The nominal mass-loss-based concentration was estimated as C_0_ = Δm/ΔV. This value does not represent compound-specific concentration; rather, it provides a consistent operational estimate for preparing and comparing smoke dilutions from different fuels.

(2) $$C_{0}=\frac{\Delta m}{\Delta V}$$

Deriving nominal concentrations from fuel mass loss is an established approach in prior combustion studies, providing a standardized, reproducible metric for total organic contaminant yield in closed-chamber systems [52]. Measuring overall relative concentration changes captures the integrated contamination burden rather than single-species dynamics [2]. The selected concentration range was chosen to span realistic fireground exposure regimes documented in previous studies, ranging from lower organic burdens characteristic of post-fire overhaul and turnout gear off-gassing to elevated smoke concentrations in enclosed spaces [12], while remaining within the dynamic range of the sensing platform.

Five exposure levels were prepared by mixing controlled smoke volumes with dry air at 22 ± 1 °C. The smoke-to-air ratio was adjusted while the total injected volume was kept constant. Temperature and humidity were maintained consistently, and each concentration was measured in triplicate.

### 3.4. Sensing Chamber and Exposure Protocol

A custom 50 mL syringe-based chamber was used to deliver diluted smoke to the coated IDE under controlled conditions. The sensor was mounted centrally so that the active surface was exposed uniformly to the chamber atmosphere. Separate inlet and outlet ports allowed smoke injection, pressure release, ventilation, and evacuation without disturbing the sensor connection. The outlet remained open during and immediately after injection to prevent pressure-induced artifacts and to promote rapid mixing. The same total volume was introduced for every measurement, and the chamber was maintained at 22 ± 1 °C and 40–50% relative humidity. Data acquisition continued until the response reached a maximum and formed a stable plateau, which was treated as the operational steady state. The outlet was then connected to a Welch WOB-L^®^ vacuum pump for a fixed regeneration interval to standardize contaminant removal between runs. Vacuum-assisted desorption was continued until the response returned to the initial operational baseline, and the subsequent exposure was initiated only after this baseline had been re-established. Separate passive-recovery tests were performed with the outlet left open for the same duration. This protocol enabled reproducible delivery, equilibration, and desorption while permitting direct comparison of vacuum-assisted and natural recovery.

### 3.5. Custom Potentiostat and Data-Acquisition System

The measurement platform combined a laboratory-built potentiostat, a PCL-818PG data-acquisition board (Advantech Co., Ltd., Taipei, Taiwan), and dedicated control software developed in Delphi^®^ 6 (Figure 2c,d). The acquisition board provided 16-bit digitization and a maximum sampling capability of approximately 110 kHz. The analog front end used two TLC074 operational amplifiers (Texas Instruments Incorporated, Dallas, TX, USA). A digital-to-analog converter generated the programmed staircase cyclic waveform, which was buffered before application to the IDE. The resulting sensor current was converted to voltage by a transimpedance amplifier with four software-selectable feedback resistors spanning 1 kΩ to 200 kΩ. Gain selection allowed weak baseline currents and larger near-saturation responses to be recorded without sacrificing resolution or exceeding the analog-to-digital (ADC) range. The control program synchronized waveform generation, gain selection, and current acquisition and allowed the user to define the initial and vertex potentials, sweep rate, potential increment, delay time, number of cycles, staircase mode, DC range, and amplifier gain. Multithreaded operation maintained sub-100 µs timing while data were displayed and processed in real time. Analog filtering was applied before digitization to suppress high-frequency circuit noise and switching transients. Together, the adjustable-gain front end and software-controlled acquisition provided the dynamic range and temporal resolution required to follow progressive uptake and release in the PDMS/f-CNT film. All raw cyclic-voltammetry current–voltage scans and the corresponding calculated differential-charge (ΔQ) values were continuously logged to a local hard drive at each scan interval, enabling both real-time monitoring and subsequent post-exposure offline analysis.

### 3.6. Continuous Cyclic-Capacitive Measurement and Signal Processing

The readout was designed to resolve small, nonstationary changes in charge storage as contaminants entered, redistributed within, and left the composite. Repeated staircase cyclic scans were applied continuously rather than measuring capacitance at one fixed bias or impedance at one frequency (Figure 2e). The 0–1000 mV window was selected from preliminary scans over broader negative-to-positive potential ranges because it provided the highest reproducible ΔQ response while maintaining stable, predominantly capacitive profiles [28]. Extending the scan below 0 mV did not improve sensitivity, whereas the positive-going window enhanced polarization and charge redistribution within the PDMS/f-CNT composite, including contributions from sorbed polarizable aromatic compounds, without appreciable leakage or irreversible signal changes. Each potential step generated a transient charging current associated primarily with the capacitive behavior of the coated IDE, superimposed on a smaller steady-state resistive component arising from the finite conductivity of the CNT-containing film.

Sequential current–potential loops were assembled into a three-dimensional dataset with potential, current, and exposure time as independent coordinates. This representation reveals both the potential regions and the time points at which the largest changes occur. Faster acquisition increases the number of scans collected during uptake and recovery and strengthens the measurable charging component, improving sensitivity to small and rapidly evolving variations in the sensing layer.

Unlike conventional electrochemical gas sensors, which typically evaluate the current at a single potential or determine capacitance under a fixed electrical condition, the present methodology continuously acquires complete cyclic voltammograms throughout the exposure process. Contaminant absorption, redistribution, and desorption continuously modify the dielectric properties of the PDMS/f-CNT layer; therefore, the electrochemical response is inherently nonstationary. Monitoring only a single current value or capacitance point may therefore overlook important transient changes or become more susceptible to baseline drift and low-frequency fluctuations. By integrating the current over the entire voltammetric cycle to obtain the transferred charge, the analytical response becomes less sensitive to localized current fluctuations while incorporating information from the complete charging process. Continuous repetition of this analysis enables simultaneous evaluation of the magnitude, rate, and temporal evolution of contaminant uptake throughout the exposure and recovery stages.

Before each smoke exposure, the same volume of clean air was introduced under identical chamber, temperature, humidity, waveform, and acquisition conditions used for smoke-containing air. The stable clean-air current–potential cycles were averaged to define the blank response. For scan k, the analytical signal, ΔQ, was calculated by subtracting the integrated charge obtained in clean air from that recorded in smoke-containing air, equivalently expressed by point-by-point subtraction of the clean-air current before integration over the selected potential interval:

(3) $$\Delta Q(k\tau )=\frac{\Delta E}{\nu }\left[\sum_{j=a}^{b}I(k,j)-\sum_{j=a}^{b}I(k_{r},j)\right]$$

or equivalently,

(4) $$\begin{array}{cccc} & \Delta Q(k\tau )=Q(k\tau )-Q(k_{r}) & & \end{array}$$

Here, k is the scan number, τ is the interval between successive scans, ΔE is the potential increment between adjacent data points, ν is the scan rate, I(k,j) is the current at point j of scan k, and I(kr,j) is the corresponding current from the averaged clean-air blank. The limits a and b define the integration window and were reordered automatically for the forward and reverse sweeps. Trapezoidal integration was used throughout. 

For an approximately capacitive response, ΔQ is related to the effective capacitance through ΔQ = CeffΔV. Accordingly, changes in the composite permittivity, interfacial polarization, and electrically accessible volume appear as changes in ΔQ. The ΔQ–time profile rises as contaminants are sorbed and retained, approaches a plateau when the uptake rate decreases, and falls during passive or vacuum-assisted desorption. Because the clean-air blank and smoke-containing air were measured using identical experimental parameters, ΔQ minimized contributions from gas injection, chamber disturbance, environmental variation, and the intrinsic electrical background. FFT processing independently suppressed broadband electronic noise, switching artifacts, and frequency components unrelated to the applied waveform. The reproducible rise, plateau, and recovery of ΔQ, together with its distinction from the clean-air and high-humidity controls, confirmed that the observed response originated from contaminant uptake rather than random noise or baseline fluctuation.

Although the model intentionally emphasizes capacitive transduction, the CNT network also introduces finite resistance. This nonideal resistive contribution affects the loop shape and steady-state current and should not be interpreted as zero. Repeatability was therefore assessed from successive sorption–desorption cycles, where deviations can arise from incomplete release, strongly retained molecules, or small differences in smoke delivery. Device-to-device reproducibility was additionally evaluated using independently coated IDEs. Humidity was examined separately because water vapor can alter the response of an unprotected IDE; the hydrophobic PDMS-rich surface was expected to limit water uptake and reduce humidity-induced charge variation. The sensor retained approximately 90% of its initial response after 20 days. Nevertheless, a protected reference IDE and temperature-assisted baseline correction will be considered in future long-term implementations to compensate for progressive resistance changes or residual-contaminant accumulation. The independently prepared PDMS/f-CNT sensors showed a device-to-device response variation of approximately 8% RSD, demonstrating good reproducibility of the fabrication and sensing response.

### 3.7. Integrated Sensing Mechanism

The complete measurement sequence can be summarized as follows. Hydrophobic and condensable fire-derived organics partition from the chamber atmosphere into the PDMS phase. Their accumulation changes polymer swelling and effective dielectric permittivity, while adsorption at f-CNT interfaces modifies local polarization and charge distribution. The IDE fringing field probes these changes, and the distributed nanotube network extends the responsive volume beyond the immediate electrode surface. Consequently, the transient charging current changes during each cyclic scan. The custom potentiostat records this current with selectable gain, FFT-assisted processing removes uncorrelated noise, and numerical integration produces ΔQ as the real-time measure of uptake and release. Thus, the chemical collection function of PDMS, the interfacial amplification provided by f-CNTs, the planar IDE geometry, and the continuous cyclic-capacitive readout operate as one coupled sensing system.

### 3.8. Surface Adsorption, Bulk Absorption, and Interfacial Polarization Mechanism

The sensing process begins when fire-derived organic molecules reach the PDMS/f-CNT coating, in which f-CNTs are distributed both near the exposed surface and throughout the polymer bulk. At the outer region of the film, accessible f-CNT surfaces can provide initial adsorption sites for smoke constituents through dispersive, hydrophobic, and π–π interactions with graphitic domains, together with complementary interactions at oxygen-containing functional groups. At the same time, hydrophobic and condensable molecules partition from the gas phase into PDMS and subsequently diffuse through the polymer, making bulk absorption within PDMS the principal mechanism for contaminant accumulation and retention. As the molecules penetrate deeper into the coating, however, their electrical detectability becomes important. In pure PDMS, molecules located close to the IDE can modify the local dielectric environment and contribute to the measured response, whereas molecules that diffuse farther into the relatively thick polymer may be only weakly sensed because PDMS is electrically insulating and provides limited electrical coupling between these deeper regions and the electrodes.

The distributed f-CNTs compensate for this limitation. CNTs embedded throughout the PDMS bulk provide additional internal adsorption interfaces for molecules that have already partitioned into the polymer, while simultaneously acting as conductive and highly polarizable domains within the insulating matrix. Consequently, the CNT–PDMS boundaries become electrically active interfaces at which charge accumulation and interfacial polarization can occur. Neighboring CNT domains separated by thin regions of dielectric PDMS can further behave as distributed microcapacitive elements, creating an electrically responsive network throughout a much larger fraction of the coating. When absorbed molecules enter these regions, changes in PDMS permittivity and swelling, together with adsorption-induced changes at f-CNT surfaces, perturb the local polarization and charge-storage behavior of the CNT–PDMS interfaces. These distributed microcapacitive and interfacial pathways therefore allow molecular uptake occurring deeper within the PDMS bulk to influence the charging current and stored charge measured at the IDE. Thus, surface-accessible f-CNTs contribute initial adsorption, PDMS provides bulk absorption and contaminant storage, embedded f-CNTs provide additional internal adsorption sites, and the distributed CNT–PDMS interfacial-polarization/microcapacitive network translates uptake throughout the coating into an enhanced electrical response.

## 4. Results

The performance of the proposed IDE/PDMS–f-CNT sensor was evaluated in terms of humidity interference, response to fire-derived smoke, signal differentiation, repeatability, and concentration-dependent behavior. The results are discussed in relation to the absorption-based sensing mechanism and the time-resolved ΔQ response.

### 4.1. Humidity Interference and Differentiation from Smoke Response

Humidity is an important interferent in capacitive gas sensing because water uptake can increase the effective permittivity and capacitance of the sensing layer, producing a charge response that may be mistaken for analyte absorption. The IDE/PDMS–f-CNT sensor was therefore exposed to approximately 80% RH, representing a substantially elevated humidity challenge relative to the 40–50% RH conditions used during the smoke experiments. For each voltammetric cycle, the current was integrated over the 0–1000 mV potential window to calculate Q. The differential charge, ΔQ, was then obtained by subtracting the average Q of the first ten baseline cycles from the charge measured in each subsequent cycle.

**Figure 3:**
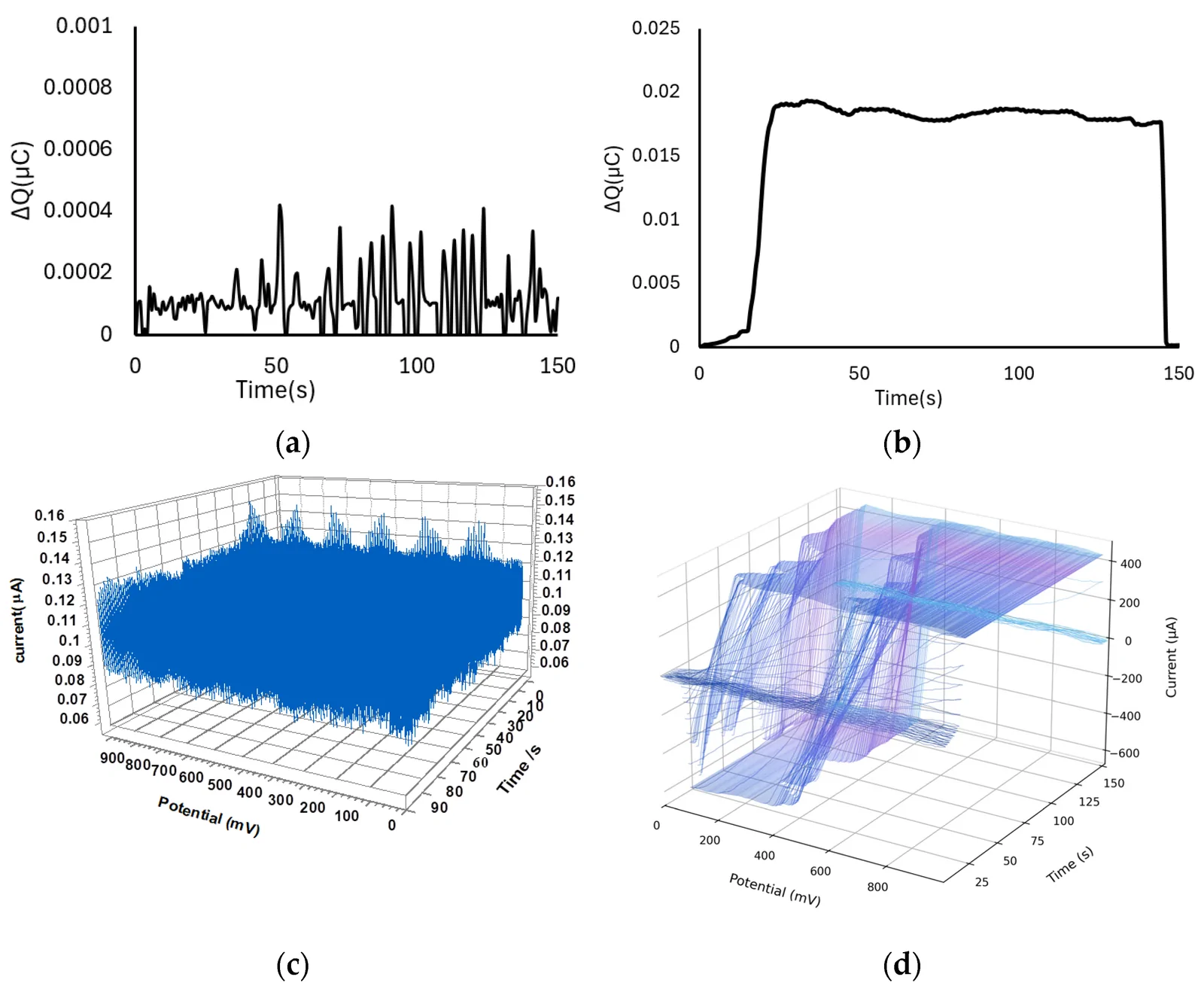
Humidity-interference evaluation at approximately 80% RH. Time-dependent ΔQ responses of (**a**) the IDE/PDMS–f-CNT sensor and (**b**) the commercial humidity sensor; corresponding three-dimensional current–potential–time profiles of (**c**) the IDE/PDMS–f-CNT sensor and (**d**) the commercial humidity sensor.

As shown in Figure 3a, the modified sensor exhibited only a small change in ΔQ, whereas the commercial humidity sensor produced a pronounced time-dependent response under the same conditions (Figure 3b). The limited response of the modified sensor is mainly attributed to the hydrophobic nature of PDMS. Its siloxane backbone is surrounded by nonpolar methyl groups that restrict water sorption and therefore limit the sensor’s sensitivity to humidity. In contrast, the hydrophilic coating of the commercial sensor readily absorbs water, increasing its effective capacitance and measured charge.

This difference is also evident in the three-dimensional voltammograms. As humidity increased, the current magnitude and enclosed CV area of the commercial humidity sensor progressively increased (Figure 3d), indicating greater capacitive charge storage following water uptake. In comparison, the IDE/PDMS–f-CNT sensor maintained a relatively stable current–potential profile and integrated CV area during humidity exposure (Figure 3c). These results demonstrate that replacing the original hydrophilic coating with the PDMS-based composite substantially reduces humidity interference, even at 80% RH.

### 4.2. Effects of f-CNT Incorporation on the Response to Paper Smoke

The contribution of f-CNTs to signal transduction was evaluated by comparing IDE/PDMS–f-CNT and IDE/PDMS sensors exposed to 200 ppm paper smoke under identical conditions. No vacuum-assisted regeneration was applied, allowing the natural uptake and desorption behavior of the two coatings to be observed. For each cycle, Q was obtained by integrating the current over 0–1000 mV, and ΔQ was calculated relative to the average charge of the first ten baseline cycles.

**Figure 4:**
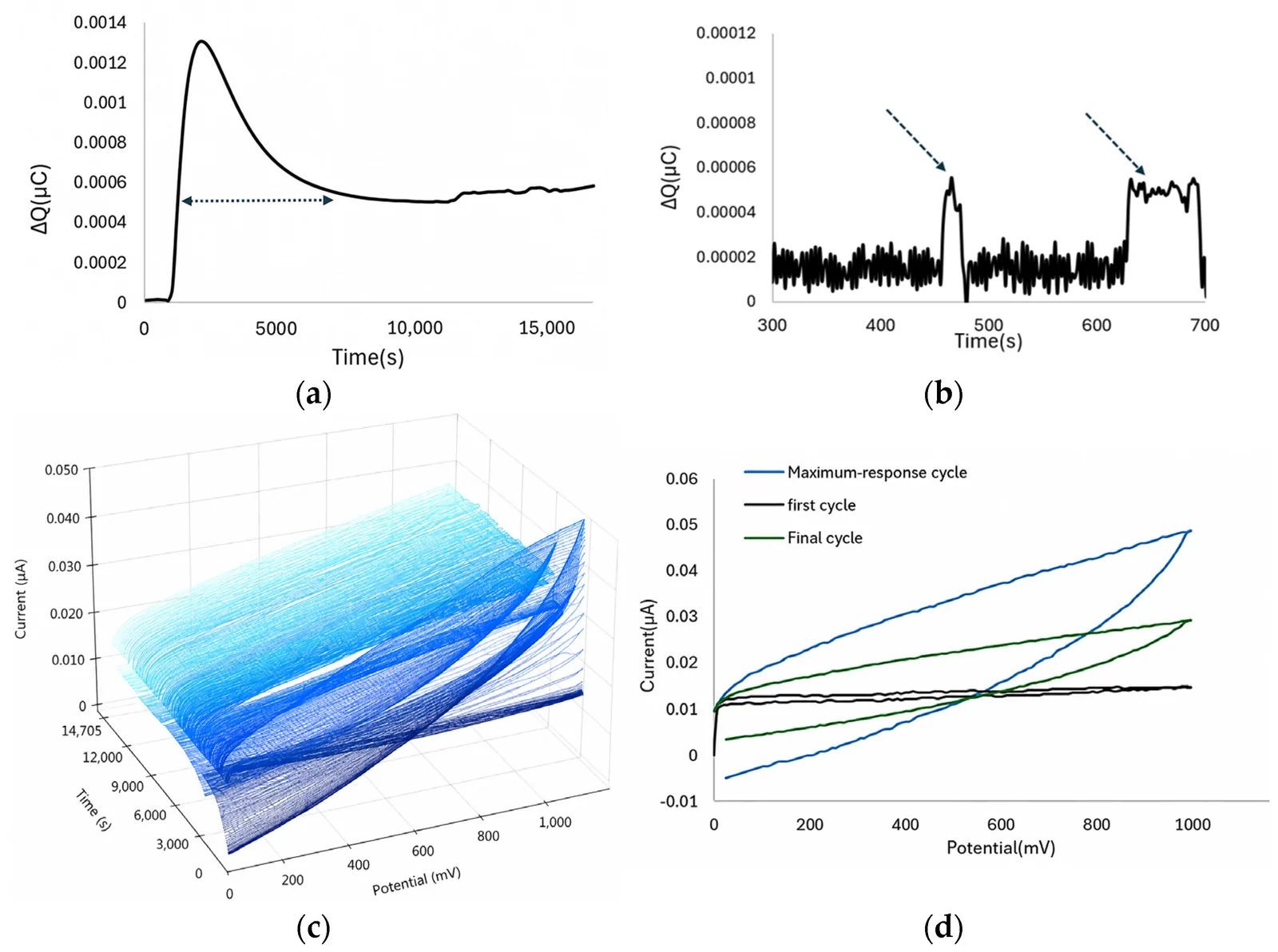
Effects of f-CNT incorporation on smoke response and natural recovery. (**a**) Time-dependent ΔQ response of the IDE/PDMS–f-CNT sensor; (**b**) time-dependent ΔQ response of the IDE/PDMS sensor during two smoke exposures; (**c**) three-dimensional current–potential–time response of the composite-coated sensor; and (**d**) representative CVs recorded at the initial, maximum-response, and final stages.

The IDE/PDMS–f-CNT sensor produced a pronounced response, reaching a maximum ΔQ of approximately $1.31\times 10^{-3}\mu C$ (Figure 4a). In comparison, the IDE/PDMS sensor exhibited only a modest response, with an average peak ΔQ of approximately $5.54\times 10^{-5}\mu C$ for the two exposure events (Figure 4b). This corresponds to an approximately 24-fold enhancement following f-CNT incorporation.

This enhancement is consistent with the proposed distributed-capacitance model. Although smoke constituents can penetrate the PDMS-only film, molecules located beyond the strongest IDE fringing-field region contribute weakly because PDMS is electrically insulating. Dispersing f-CNTs throughout the polymer introduces electrically responsive interfaces and local capacitive pathways across a larger fraction of the coating, thereby extending the effective sensing depth. The resulting evolution of the current–potential response throughout smoke uptake and natural desorption is shown in the three-dimensional voltammogram of the composite-coated sensor (Figure 4c). The larger differences among the initial, maximum-response, and final-cycle CVs further demonstrate the effect of smoke sorption on interfacial polarization, capacitance, and stored charge (Figure 4d).

The two coatings also exhibited markedly different recovery behavior. The PDMS-only response declined rapidly toward baseline, whereas the IDE/PDMS–f-CNT response decreased gradually and did not fully recover during the monitored period. Even at the lowest point of the initial post-peak decay, the composite sensor retained approximately 38% of its maximum response. More volatile or weakly sorbed constituents are expected to desorb first, whereas hydrophobic aromatic contaminants may partition more strongly into PDMS and interact with graphitic CNT surfaces, resulting in prolonged retention [31,32,33,35,37,38,39]. The effects of temperature on uptake, retention, and desorption kinetics will be investigated in future work.

### 4.3. Source-Dependent Calibration and Limit of Detection

**Figure 5:**
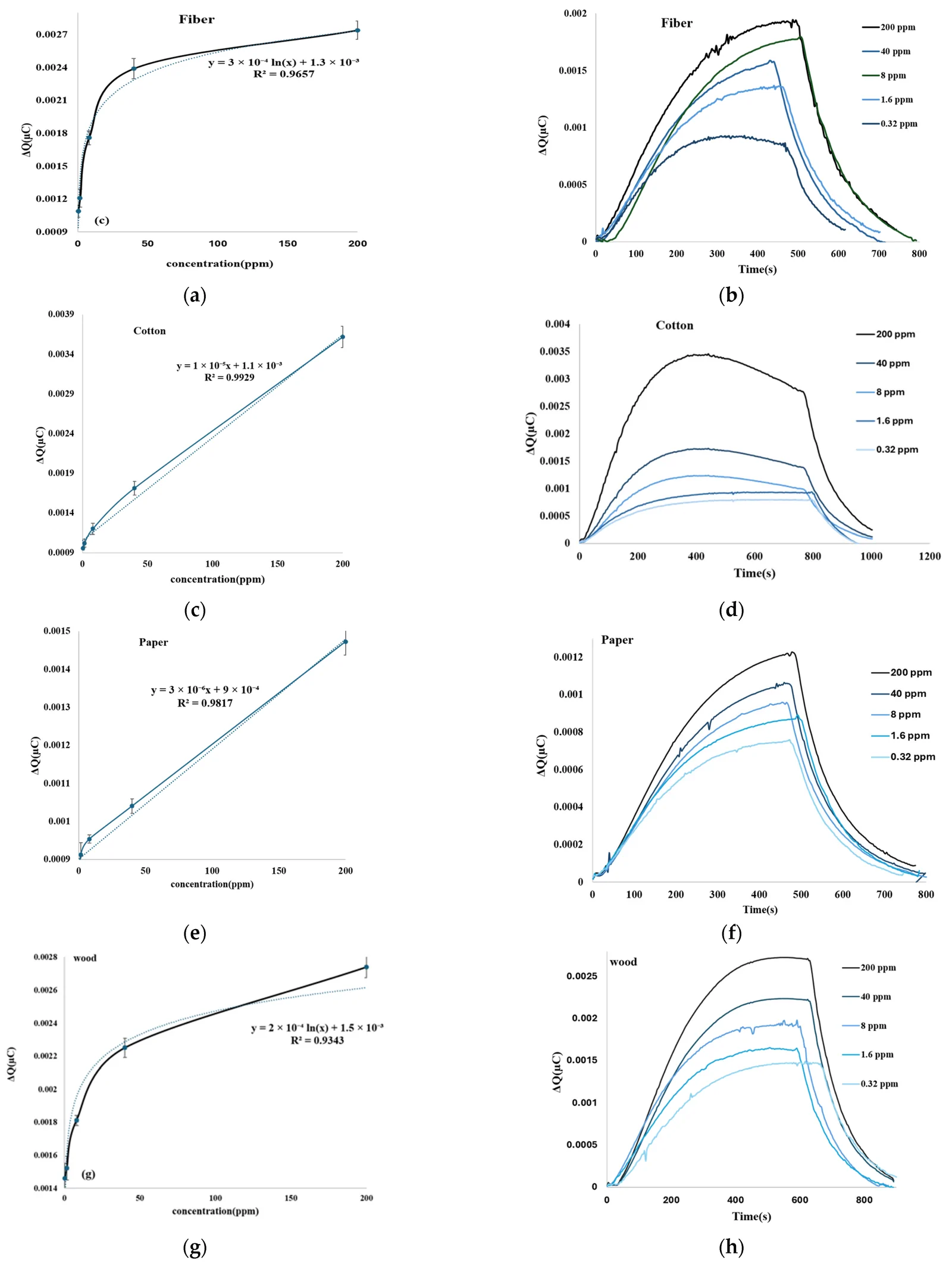
Calibration curves and corresponding ΔQ–time responses of the IDE/PDMS–f-CNT sensor for (**a**,**b**) fiber, (**c**,**d**) cotton, (**e**,**f**) paper, and (**g**,**h**) wood smoke.

The concentration-dependent response of the IDE/PDMS–f-CNT sensor was evaluated using smoke generated separately from cotton, fiber, paper, and wood (Figure 5). These materials were selected because the composition of combustion smoke is strongly dependent on the fuel source, with previous studies demonstrating source-specific differences in the relative abundance and distribution of volatile, semivolatile, aromatic, and particle-associated organic compounds [52,53,54]. For example, combustion and thermal decomposition of cotton produce a complex mixture containing light hydrocarbons, benzene, styrene, phenol, naphthalene, acenaphthylene, and phenanthrene [53], whereas wood combustion generates source-dependent distributions of VOCs, aromatic compounds, PAHs, phenolic compounds, and other semivolatile species [54]. Direct comparison of cotton, paper, and different wood materials has also demonstrated substantial differences in the concentrations and profiles of combustion-derived PAHs. Accordingly, even at the same nominal concentration, the amount partitioning into the sensing layer and the resulting electrical response were expected to depend on the combustion source. For each measurement, the charge was obtained by integrating the current over the 0–1000 mV potential window. The background-subtracted response was calculated as the difference between the charge measured after smoke introduction and the average charge of the first ten pre-exposure cycles. To standardize the experimental conditions, vacuum-assisted desorption was applied for a fixed period after each exposure to remove retained contaminants and return the sensor toward a comparable baseline before the next measurement. Each experiment was repeated three times, and the calibration responses are presented as mean ± standard deviation.

As shown in Figure 5, increased with nominal smoke concentration for all four materials, although the magnitude and shape of the calibration curves differed. Some sources exhibited an approximately linear response, whereas others showed nonlinear or saturation-type behavior at higher concentrations. These differences are attributed to variations in combustion composition and in the volatility, condensability, molecular size, aromaticity, and sorption affinity of the resulting constituents toward the PDMS/f-CNT layer. Previous GC–MS and FTIR studies of different fire sources have similarly identified source-specific chemical markers and distinct smoke compositions [42]. Progressive occupation of accessible sorption regions may also reduce the incremental response at elevated concentrations.

Ten independent fresh-air blank measurements (n=10) were performed in the absence of smoke under the same injection, chamber, environmental, acquisition, filtering, and charge-integration conditions used for the smoke-containing samples. Thus, the only controlled difference was the absence or presence of smoke-derived contaminants. The standard deviation of the resulting blank ΔQ responses was defined as $\sigma_{blank}$. For each combustion source, the LOD was calculated using the slope obtained from the corresponding linear calibration range according to the following:

(5) $$LOD=\frac{3\sigma_{blank}}{m}$$

Here, m  is the slope of the linear regression within the selected linear range. The corresponding linear range ($R^{2}$), calibration slope (m), and calculated LOD values are summarized in Table 2. Differences among the LOD values reflect source-dependent sensitivity arising from variations in smoke composition, volatility, condensability, molecular size, aromaticity, and sorption affinity toward the PDMS/f-CNT layer.

### 4.4. Cumulative Exposure Tracking

**Figure 6:**
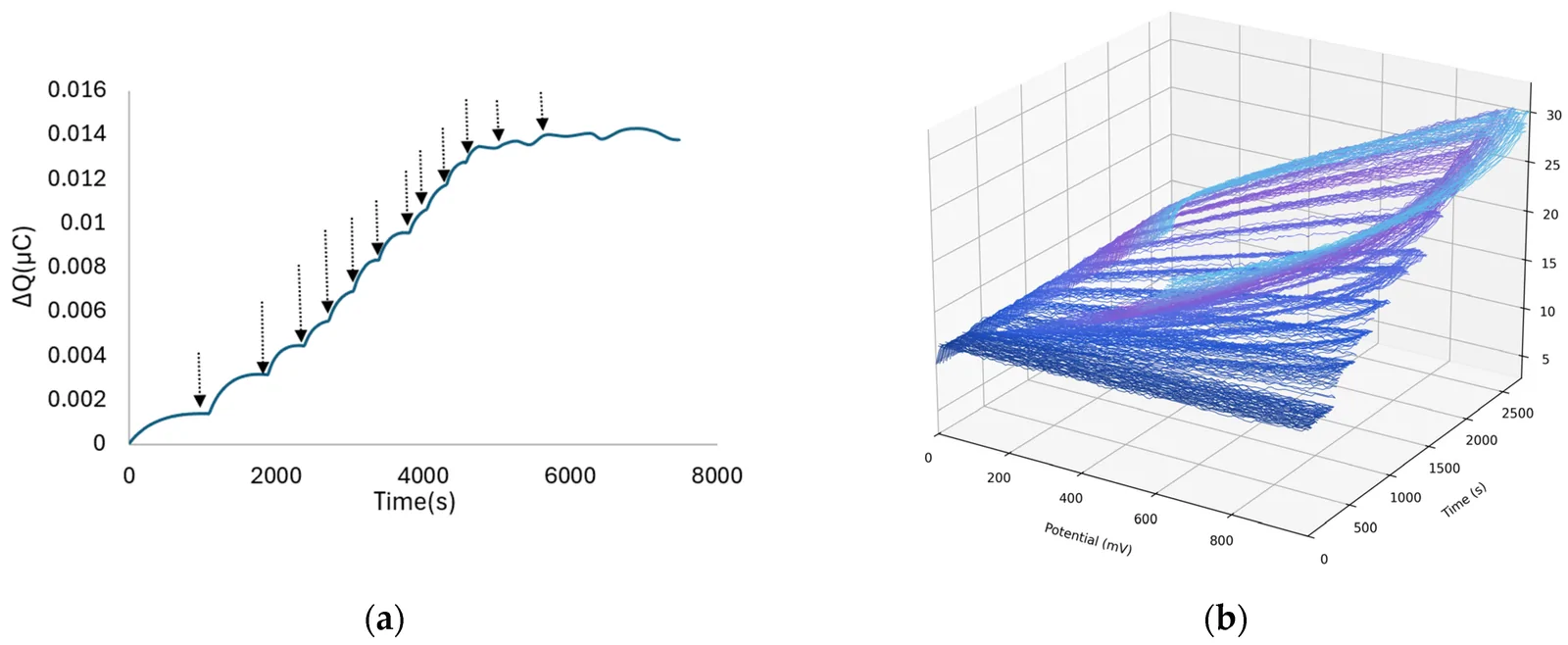
Cumulative mass-capacitive response during sequential smoke loading. (**a**) Stepwise ΔQ response to successive injections; (**b**) corresponding three-dimensional current–potential–time profiles.

To investigate whether the sensor could preserve exposure history while continuing to register newly introduced contamination, equal smoke aliquots were sequentially injected into the chamber without evacuation or regeneration between exposures (Figure 6a). Each smoke addition produced a further increase in ΔQ, while the signal remained elevated during the intervals between injections. Sorption into the PDMS/f-CNT layer is an equilibrium-controlled process described by the relationship between gas-phase concentration, equilibrium uptake, and effective surface coverage. Each smoke injection temporarily increased the contaminant concentration in the chamber, establishing a concentration gradient that drove further uptake into the sensing layer. Because no evacuation or regeneration was performed between injections, contaminants retained from previous exposures continued to contribute to the measured charge. The response to each newly introduced smoke dose was therefore superimposed on the residual contribution of previous exposures, producing the observed staircase-like profile. During the nearly horizontal interval following each step, the sensing layer approached equilibrium with the prevailing chamber concentration, and further net uptake became limited. The corresponding three-dimensional current–potential–time profiles (Figure 6b) further demonstrate the progressive evolution of the capacitive response as contaminants accumulate within the sensing layer. 

This cumulative behavior is particularly important for the mass-capacitive concept because the sensor acts as both a contaminant-collecting layer and an electrical transducer. Rather than responding only to the instantaneous gas concentration, the retained mass of absorbed smoke constituents produces a persistent capacitive signal that represents the integrated exposure history. This exposure-history retention is particularly relevant to firefighter monitoring because firefighters may move between environments with substantially different contamination levels, including active fire zones, exterior operations, overhaul areas, apparatus cabins, and fire stations. A sensor that measures only the instantaneous atmosphere may fail to represent the combined burden accumulated across these locations. In contrast, the response observed here suggests that the proposed platform can integrate sequential contamination events and provide a running indication of cumulative smoke exposure.

At higher cumulative loading, however, the incremental response to subsequent injections decreased, and ΔQ approached a plateau near 0.014 μC. The progressive decrease in step height reflects the evolution of the sorption isotherm with increasing loading and surface coverage [55,56]. During the initial injections, the effective surface coverage was low and many readily accessible sorption regions remained available. Uptake was therefore consistent with the initial region of a Langmuir-type isotherm, where each increase in gas-phase concentration produces a comparatively large increase in coverage and sorbed mass. Accordingly, the first smoke additions generated relatively large increases in ΔQ.

As sequential exposure continued, the surface coverage and contaminant concentration within the composite increased. Fewer readily accessible regions remained, and the concentration gradient driving further uptake progressively decreased. Heterogeneity among sorption regions, occupation of domains with different affinities, and interactions among retained molecules also became more influential, producing increasingly Freundlich-like sorption behavior at higher coverage. Each subsequent injection therefore generated a smaller increase in equilibrium uptake and a smaller ΔQ step. The plateau near 0.014 μC indicates that the sensing layer approached an operational sorption equilibrium under the applied conditions rather than undergoing an abrupt loss of sensitivity.

Because sorption is reversible, accumulated contaminants may gradually desorb as the surrounding concentration decreases. As demonstrated by the natural-recovery response in Figure 4, this process may require an extended period, particularly for compounds with strong affinity for the PDMS/f-CNT layer. More volatile or weakly retained constituents are expected to be released earlier, whereas strongly retained hydrophobic and semivolatile contaminants may remain longer [57]. The persistent response may therefore become weighted toward high-affinity compounds, potentially including PAHs relevant to carcinogenic firefighter exposure, although the sensor does not selectively identify individual carcinogens. Approximately 38% of the maximum response remained during the monitored natural-recovery period, confirming the prolonged retention of part of the absorbed mixture. 

When rapid regeneration is required between independent measurements, desorption can be accelerated by replacing the contaminated atmosphere with fresh air or applying reduced pressure. In the present calibration experiments, vacuum-assisted regeneration was performed manually and restored the sensor response to its operational baseline before the next exposure. In future field-oriented systems, this procedure could be automated by integrating a fresh-air or vacuum pump with electronically controlled valves, enabling repeated sampling, regeneration, and baseline verification over extended monitoring periods.

## 5. Discussion

The experimental findings presented in Section 4 establish the functionality of the proposed absorption-based mass-capacitor. Unlike many portable gas sensors designed for a single compound or a narrow chemical group, this platform targets a broader fraction of hydrophobic and condensable organic contaminants in smoke, including hazardous aromatic VOCs and PAH-related compounds. The sensor showed low humidity interference, while f-CNT incorporation markedly increased the measured response. It also responded to changes in smoke concentration and continuously tracked repeated exposure events over time. The sensing response arises from the complementary functions of PDMS and f-CNTs. Hydrophobic and condensable organic compounds partition into the PDMS layer, while aromatic molecules can interact with graphitic CNT surfaces through hydrophobic and π–π interactions. Their accumulation changes the dielectric and charge-storage properties of the composite. The distributed f-CNT network improves electrical coupling, interfacial polarization, and charge redistribution, allowing absorption throughout a larger portion of the coating to contribute to the measured ΔQ. Continuous cyclic measurements therefore provide real-time information about contaminant uptake, accumulation, retention, and release, rather than only an instantaneous gas-phase response. At this stage, the primary objective was to establish the ability of the proposed mass-capacitor to sorb the organic fraction of smoke and convert the accumulated contamination into a measurable electrical response. The experiments were therefore performed in a closed chamber under controlled laboratory conditions. This configuration minimized uncontrolled airflow, temperature fluctuations, and direct particle deposition, allowing the intrinsic sorption and electrical response of the sensing layer to be evaluated reproducibly. The current signal represents the overall accumulated contamination burden and does not yet identify individual compounds within the smoke mixture.

Future studies will evaluate the platform under more demanding and realistic conditions, including elevated and changing temperatures, open-flow environments, variable humidity and airflow, soot deposition, and more complex smoke mixtures. For field-oriented implementation, the sensing element could be housed within a compact shielded chamber to reduce rapid environmental fluctuations. In addition, a replaceable, size-selective, gas-permeable inlet filter could limit direct deposition of coarse soot while allowing gaseous and smaller semivolatile contaminants to reach the PDMS/f-CNT layer. The effects of soot loading, airflow, and filter characteristics on sensitivity, response kinetics, and long-term stability will be systematically evaluated. Future studies will also expand the calibration library to include a broader range of individual fuel sources and mixed-source combustion scenarios, combined with GC–MS characterization to determine how source composition and fuel combinations influence the mass-capacitor response, and to support the development of a multi-source calibration strategy. These investigations will help define the operating range, maximum loading capacity, long-term stability, retention behavior, and regeneration requirements of the sensing layer. Controlled temperature-programmed desorption may also provide additional information about the retained contaminant mixture. Because individual compounds differ in volatility and affinity for PDMS and CNT surfaces, weakly retained compounds may be released first, while more strongly retained compounds may require higher temperatures for desorption. Inspired by gas chromatographic separation, the order and rate of release could generate characteristic affinity-based response patterns for partial discrimination among contaminant groups. This concept will require systematic validation using controlled compounds and defined mixtures. Combining multiple sorptive coatings with different affinities may further improve discrimination and support future portable or wearable monitoring of cumulative firefighter exposure.

## 6. Conclusions

This work demonstrates a novel absorption-based PDMS/f-CNT mass-capacitor for continuous monitoring of fire-derived organic contamination. By integrating sorptive accumulation and electrical transduction within a single sensing layer, the platform converts contaminant uptake, retention, and release into a time-resolved differential-charge response, enabling cumulative exposure tracking rather than measurements of only the instantaneous concentration of selected airborne species. Incorporation of f-CNTs increased the response by approximately 24-fold compared with PDMS alone, confirming the importance of the distributed CNT network for enhanced electrical transduction of contaminant uptake. The PDMS-rich composite also showed minimal interference at approximately 80% RH, supporting its ability to suppress water-induced responses under elevated humidity conditions. The sensor produced clear concentration-dependent responses to smoke generated from cotton, fiber, paper, and wood, with source-dependent calibration behavior reflecting differences in smoke composition and sorption affinity. Sub-ppm detection limits were achieved across the investigated combustion sources. Sequential smoke exposures produced progressive stepwise increases in response, demonstrating the capability of the mass-capacitor to follow cumulative contaminant loading. During natural recovery, approximately 38% of the maximum response remained, indicating retention of a higher-affinity fraction of the accumulated contamination and demonstrating the ability of the sensing layer to preserve exposure history after the external contamination level decreased. The sensor further retained approximately 90% of its initial response after 20 days, providing preliminary evidence of short-term stability under the tested conditions. Collectively, these results establish the feasibility of the PDMS/f-CNT mass-capacitor as a continuous sorptive sensing platform under controlled laboratory conditions. The present results should not yet be interpreted as validation of performance under actual fireground conditions. Future work will therefore evaluate the platform under variable temperature, humidity, airflow, soot loading, and mixed-source smoke environments and correlate the electrical response with chemical analysis, such as GC–MS. Controlled desorption, improved regeneration, and multiple sorptive phases will also be investigated to extend the platform toward contaminant discrimination and eventual portable or wearable exposure-monitoring applications.

## Figures and Tables

**Table 2:** Calibration and detection performance of the IDE/PDMS–f-CNT sensor for different smoke sources.

Smoke Source	LOD (ppm)	R^2^	Slope, m
Cotton	0.17	0.9929	1.0 × 10^−5^
Fiber	0.05	0.9921	3.0 × 10^−5^
Paper	0.26	0.9817	3.0 × 10^−6^
Wood	0.08	0.995	2.0 × 10^−5^

## Data Availability

The original contributions presented in this study are included in the article. Further inquiries can be directed to the corresponding author.

## Abbreviations

The following abbreviations are used in this manuscript:

ADC	Analog-to-digital converter
ANN	Artificial neural network
CNT	Carbon nanotube
CV	Cyclic voltammetry
DC	Direct current
f-CNT	Functionalized carbon nanotube
FFT	Fast Fourier transform
FTIR	Fourier-transform infrared spectroscopy
GC–MS	Gas chromatography–mass spectrometry
IDE	Interdigitated electrode
KPS	Potassium persulfate
LOD	Limit of detection
MOF	Metal–organic framework
MOX	Metal-oxide semiconductor
MWCNT	Multi-walled carbon nanotube
PAH	Polycyclic aromatic hydrocarbon
PCA	Principal component analysis
PDMS	Polydimethylsiloxane
PEG	Polyethylene glycol
RH	Relative humidity
SCBA	Self-contained breathing apparatus
SWCNT	Single-walled carbon nanotube
TVOC	Total volatile organic compound
VOC	Volatile organic compound
